# Semaglutide as a potential therapeutic adjunct for reducing flare-ups in Sjögren’s syndrome: A case report

**DOI:** 10.1177/2050313X251372813

**Published:** 2025-08-31

**Authors:** John Jung, Ismail Zazay, Priya Kalia, Neda Shaghaghi, Nancy Ross, Benjamin Homan

**Affiliations:** 1 John Sealy School of Medicine, The University of Texas Medical Branch, Galveston, TX, USA; 2 Department of Family Medicine, The University of Texas Medical Branch, Galveston, TX, USA

**Keywords:** Sjögren’s syndrome, GLP-1 receptor agonist, semaglutide, autoimmune disease, weight loss, case report

## Abstract

Sjögren’s syndrome is a chronic autoimmune disorder with limited therapeutic options. Glucagon-like peptide-1 (GLP-1) receptor agonists, primarily used for diabetes and obesity, have emerging evidence of anti-inflammatory and immunomodulatory effects. We present a case of a 37-year-old woman with primary Sjögren’s syndrome and comorbid Hashimoto’s thyroiditis and seronegative rheumatoid arthritis, who experienced frequent debilitating flares affecting her quality of life. After starting semaglutide for weight management and losing over 100 lbs, the patient reported a dramatic reduction in flare frequency and duration, with episodes decreasing from multi-week flares to approximately a single day. This case suggests that semaglutide may offer dual benefits in Sjögren’s syndrome by combining substantial weight reduction with possible direct immunomodulatory effects. Further research is warranted to explore GLP-1 agonists as adjunctive therapy in the management of autoimmune diseases.

## Introduction

Sjögren’s syndrome is a systemic autoimmune disease primarily characterized by lymphocytic infiltration of exocrine glands, leading to keratoconjunctivitis sicca and xerostomia. It affects an estimated 0.1%–0.6% of the population, disproportionately impacting women. The disease is frequently associated with systemic symptoms, including fatigue, joint pain, and vasculitis. Although classically considered a disease of adults, Sjögren’s syndrome can also present in pediatric and adolescent patients.^1^

Conventional management remains palliative, focusing on symptom control with artificial tears and saliva substitutes, and in some cases, systemic immunosuppressants. However, many patients report ongoing disability and diminished quality of life due to incomplete response and side effects of current therapies. This therapeutic gap underscores the need for novel agents with both metabolic and immune-regulatory potential.

Glucagon-like peptide-1 (GLP-1) receptor agonists, such as semaglutide, have well-documented roles in obesity and glycemic control. More recently, attention has shifted toward their anti-inflammatory and immunomodulatory effects, including downregulation of NF-κB pathways, suppression of IL-6 and TNF-α, and enhancement of regulatory T-cell responses.^2,3^ These mechanisms warrant exploration of GLP-1 analogues in autoimmune conditions, including Sjögren’s syndrome.

## Case presentation

A 37-year-old female with a history of primary Sjögren’s syndrome, Hashimoto’s thyroiditis, seronegative rheumatoid arthritis, hypertension, and anxiety presented with chronic, burdensome autoimmune symptoms. Prior to treatment with semaglutide, she experienced flare-ups every few weeks characterized by severe fatigue, widespread arthralgia, dryness of the eyes and mouth, and severe malaise. She described these episodes as “completely draining,” often leaving her bedridden for an entire day or longer.

Her medical regimen included levothyroxine 100 mcg daily, duloxetine 60 mg daily, propranolol 10 mg daily, and lisinopril-HCTZ 20-12.5 mg daily. Despite stable thyroid and blood pressure control, her autoimmune symptoms persisted. At baseline, her weight was 291 lbs (body mass index >40), and she expressed significant frustration over both her physical health and limited treatment options.

**Figure 1.**
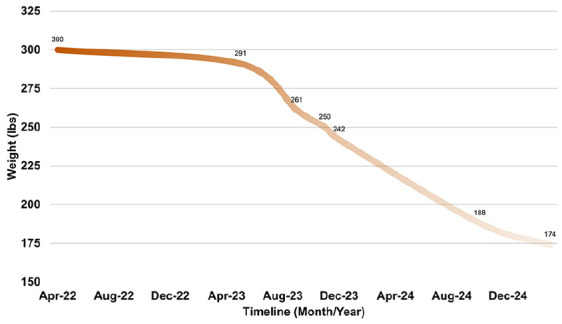
Weight trend and clinical symptom improvement post-semaglutide. The *x*-axis shows time (months) since semaglutide initiation, and the *y*-axis shows weight in pounds (lbs). Shaded regions indicate periods of Sjögren’s symptom flare-ups, illustrating their reduction during the weight loss trajectory. Gradient shading is for visual emphasis only and not a quantitative measure.

In early 2023, she initiated semaglutide (Wegovy), titrated up to 2.4 mg weekly. Over the course of 6 months, she lost a total of 117 lbs, reaching a weight of 174 lbs. More notably, she reported a near-complete resolution of Sjögren’s flares. Episodes became infrequent and brief, typically resolving within a day without new interventions. She described feeling “like a completely different person,” attributing her improvement to both weight loss and the systemic impact of semaglutide (Figure 1 illustrates this weight reduction and symptom improvement).

## Discussion

The resolution of chronic flares in this patient following semaglutide initiation suggests possible immunologic changes. GLP-1 receptor agonists such as semaglutide are increasingly recognized for pleiotropic effects beyond glycemic control, particularly their anti-inflammatory and immune-modulating properties.^2,3^ Preclinical and human studies have demonstrated that semaglutide reduces systemic markers of inflammation, including IL-6, TNF-α, and C-reactive protein, and can suppress pro-inflammatory macrophage activity.

Adipose tissue is now recognized not just as an energy store, but also as an active endocrine organ contributing to chronic low-grade inflammation. In obesity, hypertrophic adipocytes release a spectrum of pro-inflammatory adipokines (e.g., leptin and resistin) and cytokines (e.g., IL-1β and IL-6), which are known to exacerbate autoimmune conditions. Weight loss itself, therefore, has been linked to decreased autoimmune activity.^3,4^ It is plausible that both semaglutide-induced metabolic improvements and its direct immunologic effects synergistically contributed to the patient’s improvement.

Moreover, recent mechanistic insights have added further intrigue. Nakamura et al.^5^ reported that Sjögren’s glandular dysfunction may stem from a lysosome-dependent cell death pathway involving impaired autophagic degradation of caspase-8. While not directly tested in Sjögren’s, semaglutide has been implicated in upregulating autophagic and lysosomal activity in other contexts, suggesting a possible protective mechanism at the glandular level. This hypothesis remains speculative, but it opens a new line of investigation into the molecular overlap between metabolic and autoimmune pathways.

Lastly, the advent of dual and tri-agonist therapies (e.g., tirzepatide and retatrutide) targeting GLP-1, GIP, and glucagon receptors may further enhance anti-inflammatory outcomes. These drugs may eventually prove superior to semaglutide in autoimmune modulation. As such, this case underscores the need for clinical trials evaluating GLP-1 analogues in autoimmune conditions.

Despite this promising outcome, we acknowledge several limitations. A primary limitation of this case is the impossibility of disentangling the profound anti-inflammatory benefits of substantial weight loss from a potential, direct immunomodulatory effect of semaglutide. Future studies with control groups will be necessary to isolate these variables. It should also be noted that the assessment of disease activity was based solely on patient-reported outcomes, with no formal disease activity indices (e.g., ESSDAI and ESSPRI) or objective glandular measurements documented. Future prospective studies should aim to correlate these subjective improvements with objective markers, such as inflammatory cytokines (e.g., IL-6 and TNF-α), autoantibody levels, or functional tests of salivary gland output. In addition, the patient’s constellation of autoimmune comorbidities (Hashimoto’s thyroiditis and seronegative rheumatoid arthritis) may limit the generalizability of this single-case observation.

## Conclusion

This case highlights a remarkable clinical response to semaglutide in a patient with Sjögren’s syndrome, marked by dramatic weight loss and substantial reduction in flare frequency and duration. The observed outcomes may reflect a combination of metabolic, immunomodulatory, and possibly lysosome-stabilizing effects. While anecdotal, this report invites further exploration of GLP-1 agonists as adjunct therapies in autoimmune diseases. Future studies should explore mechanistic pathways and identify patient populations most likely to benefit.
